# Rapid growth rate of *Enterobacter sp*. SM3 determined using several methods

**DOI:** 10.1186/s12866-024-03547-3

**Published:** 2024-10-10

**Authors:** Sophie Pollack-Milgate, Sanchi Saitia, Jay X. Tang

**Affiliations:** Department of Physics, Brown University, 182 Hope Street, Providence, RI 02912 USA

**Keywords:** Exponential growth, Doubling time, Growth rate, Activation phase, Lag phase, Colony-forming unit, Plaque forming unit, Optical absorbance, Enterobacteria, Gut microbiome

## Abstract

**Background:**

Bacterial growth rate, commonly reported in terms of doubling time, is frequently determined by one of two techniques: either by measuring optical absorption of a growing culture or by taking samples at different times during their growth phase, diluting them, spreading them on agar plates, incubating them, and counting the colonies that form. Both techniques require measurements of multiple repeats, as well careful assessment of reproducibility and consistency. Existing literature using either technique gives a wide range of growth rate values for even the most extensively studied species of bacteria, such as *Escherichia coli*, *Pseudomonas aeruginosa*, and*  Staphylococcus aureus*. This work aims to apply several methods to reliably determine the growth rate of a recently identified species of *Enterobacteriaceae*, called *Enterobacter sp. SM3*, and to compare that rate with that of a well-known wildtype *E. coli* strain KP437.

**Results:**

We extend conventional optical density (OD) measurements to determine the growth rate of *Enterobacter sp. SM3*. To assess the reliability of this technique, we compare growth rates obtained by fitting the OD data to exponential growth, applying a relative density method, and measuring shifts in OD curves following set factors of dilution. The main source of error in applying the OD technique is due to the reliance on an exponential growth phase with a short span. With proper choice of parameter range, however, we show that these three methods yield consistent results. We also measured the *SM3* division rate by counting colony-forming units (CFU) versus time, yielding results consistent with the OD measurements. In lysogeny broth at 37^o^C, SM3 divides every 21 ± 3 min, notably faster than the RP437 strain of *E. coli*, which divides every 29 ± 2 min.

**Conclusion:**

The main conclusion of this report is that conventional optical density (OD) measurements and the colony-forming units (CFU) method can yield consistent values of bacterial growth rate. However, to ensure the reproducibility and reliability of the measured growth rate of each bacterial strain, different methods ought to be applied in close comparison. The effort of checking for consistency among multiple techniques, as we have done in this study, is necessary to avoid reporting variable values of doubling time for particular species or strains of bacteria, as seen in the literature.

## Background

The range of variation in bacterial growth rate is wide, with some bacteria being dormant or extremely slow-growing and others dividing as fast as every 20 to 30 min. During an exponential growth phase, bacterial growth rate is mathematically defined by

1 $$k=\frac{T_{d}}{log2},$$

where *k* is the rate constant and $\:{T}_{d}$ is the doubling time, which is equal to the average time of division [1]. Under favorable conditions, a bacterial culture can reach a density of several billion cells per milliliter in a nutrient-rich liquid medium. The density of bacteria can reach much higher values in certain regions, such as in dense colonies or swarms on an agar surface [2, 3], or when the bacteria form biofilms [4]. Exploring and comparing the growth rates and densities that bacteria attain under a range of conditions provides insights into their metabolism and physiology [5, 6]. Such studies are broadly important, with applications encompassing environmental microbiology [7], food preservation [8, 9], infection control [10], etc. Therefore, a variety of techniques and methods have been developed to measure bacteria growth rate.

The most convenient technique for determining bacterial concentration is to measure optical absorption [11]. This technique relies on the Beer-Lambert law, which predicts a proportional relationship between optical absorbance – also known as optical density (OD) – and the concentration of particles that absorb or scatter light on its path. When applied to measuring the density of bacteria, Beer’s law holds at relatively low density, in the so-called exponential phase of the growth. OD is defined as the logarithm in the base of ten of the ratio of incident light to transmitted light. When over 90% of incident light is absorbed or scattered, i.e., less than 10% of the light is transmitted (OD > 1.0), the OD readings are no longer proportional to the density of bacteria due to multiple scattering events of light on its path [12]. As a result, this method of determining bacterial concentration becomes inaccurate at high densities of bacteria.

Several derivative approaches rely on the same principle of optical absorption. For example, one can use plate readers to record optical absorbance instead of using conventional spectrometric cells [12, 13]. Instead of following the individual growth curves, one can also measure the so-called start of growth time (SGT) [14]. Another modified approach to the optical density measurement is the relative density method [15], which can yield bacterial doubling time even if the OD readings deviate from their proportional relationship with cell density.

An independent, century-old technique measuring the density of dividing bacteria is to count colony-forming units (CFUs) on agar plates following serial dilution, inoculation, and incubation [16, 17]. This technique, although slow and labor-intensive, is reliable because it is based on directly counting live cells. Modifications of this technique include counting colonies per droplet spread [18, 19] or per track [20], using a microplate reader [21], or taking images using a digital camera [22]. Alternative methods have also been developed, such as counting cells under an optical microscope or using a flow cytometer to count cells (FACS) [23]. In contrast to counting colonies on agar plates, modified methods have not gained wide use, perhaps because they typically require equipment that is not routinely available in most microbiology labs.

A recently identified species in the family of *Enterobacteriaceae*, *Enterobacter sp SM3*, has sparked much interest in the area of gut physiology [24]. SM3 was isolated from the feces of mice suffering from dextran sodium sulfate (DSS) induced dysbiosis, which is broadly associated with intestinal disorders [25]. SM3 has been found in the inflamed colon of patients who suffer from inflammatory bowel disease (IBD), and it has been shown to ameliorate inflammation in colitic mice [24]. The basic properties of *SM3*, such as its fast proliferation rate and its swarming motility, have been implicated in its physiological role in reducing intestinal inflammation [24]. Thus, it is important to reliably determine the growth rate of this new species of *Enterobacteriaceae*.

In this study, we apply both OD measurements and the CFU method to reliably determine the growth characteristics of SM3 in close comparison with a well-known wild type strain of *E. coli*, RP 437. For the OD technique, in particular, we undertake several approaches to assess its reliability. The consistent results among all test methods confirm the validity of these methods. Limitations of the OD-based methods at either very low or very high concentrations are remedied by the CFU method, which has its own limitation of counting only live and dividing cells. This study’s contribution is in providing an example of applying and comparing different methods of growth rate determination to confirm their consistency and overcome particular limitations of individual methods. Thus, this study provides broadly applicable guidance on reducing variability in measurements of the bacterial growth rate and doubling time.

## Results and discussion

### A log-linear plot of OD versus time shows an exponential phase of the cell growth, followed by slowed growth towards saturation

**Fig. 1:**
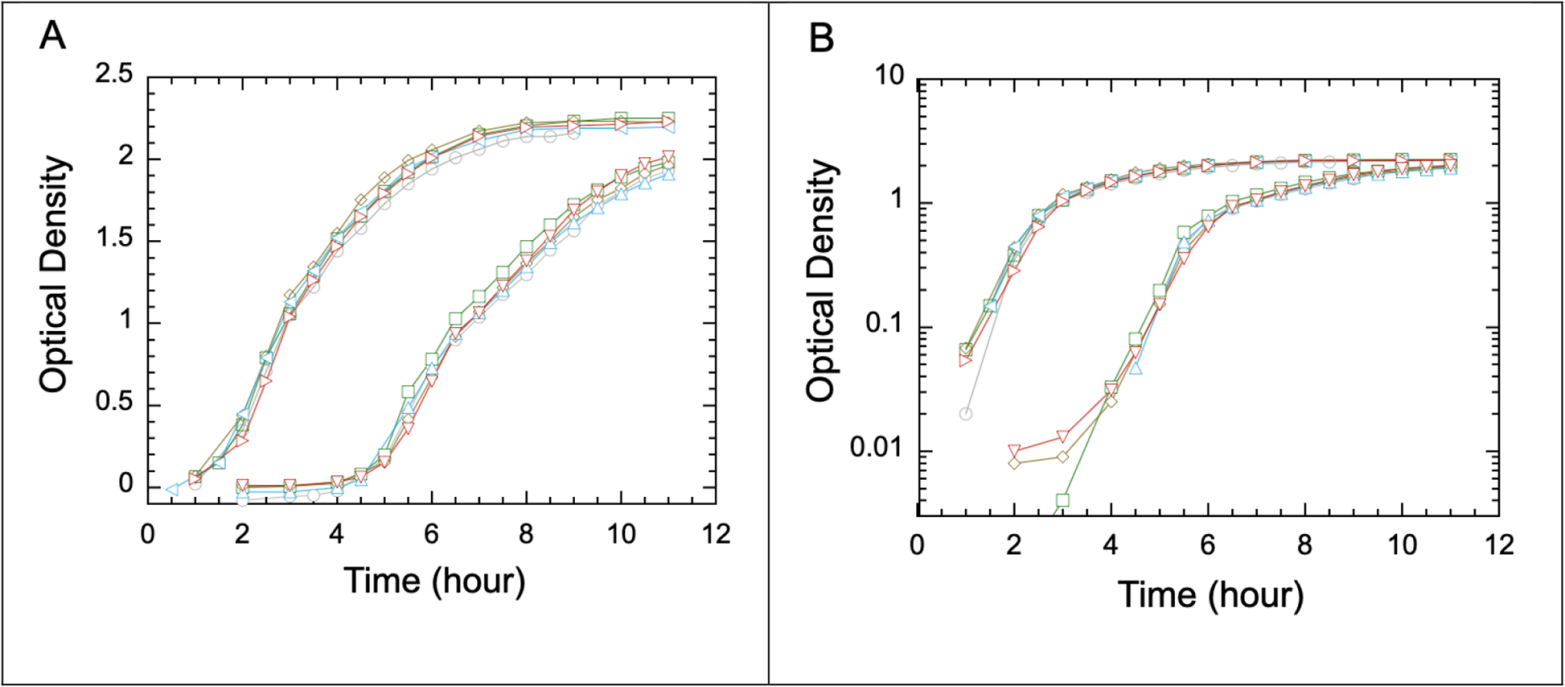
Growth of SM3 detected by measurements of optical density (OD) as a function of time. The data are plotted first on a linear-linear scale (**A**) and then a log-linear scale (**B**). All curves show an exponential phase followed by slower growth towards saturation. Five repeated measurements confirm good reproducibility. Within the same figure, the set of curves on the left are measurements of fresh growth cultures, and those on the right are regrowth cultures, each following a 1:1000 dilution from a fresh growth at the 1-hour mark. The bacterial growth rate can be obtained from the slope of the linear section of the log-linear plot, as shown in Fig. 2

To avoid complications from a known lag or activation phase that follows a fresh growth from an overnight culture, we designed a measurement procedure that uses a two-stage sequence of bacterial growth. The first set of measurements was performed on samples diluted from an overnight culture by a factor of 1:200 (Fig. 1, curves on the left), which we call the fresh growth. One hour into the fresh growth, samples were taken and diluted by 1:1000 in pre-warmed LB, creating a new growth called a regrowth. The OD values of the regrowth samples were measured, with results shown as the curves on the right. When the data are shown in log-linear plots (Fig. 1B), the range of exponential growth is clearly discernible. This range, a narrow interval of under two hours, is typically relied upon for a fit to obtain the bacterial growth rate. While the slopes of the two sets of curves are visibly the same, those from the second set of curves, the regrowth, are more accurate for obtaining the cell growth rate for two reasons. First, unlike the curves for fresh growth, the regrowth takes place following the activation period, which typically lasts about one hour. Thus, the cells grow exponentially from the start of regrowth, yielding the growth rate more reliably. Second, since the bacterial growth curves deviate notably from exponential behavior as the OD value surpasses 0.5 or even a bit lower [6], the lower starting OD values of the regrowth curves yield a longer range of data that follow a linear slope in the log of OD versus time plot than that for the fresh growth curves. This improvement due to the second dilution step to start a regrowth is particularly significant when measuring species of rapidly growing bacteria such as the *Enterobacter* sp. *SM3*, which is the main subject of this study. Using a standard spectrophotometer measuring a bulk sample of 1.0 cm path length, we note that the reliable exponential growth range is between OD values of 0.05 and 0.5. Bearing this practical range in mind, the two-step dilution design may be substituted with a single dilution of a large enough factor or by direct inoculation from a frozen stock with only a small number of bacteria introduced into the liquid medium.

**Fig. 2:**
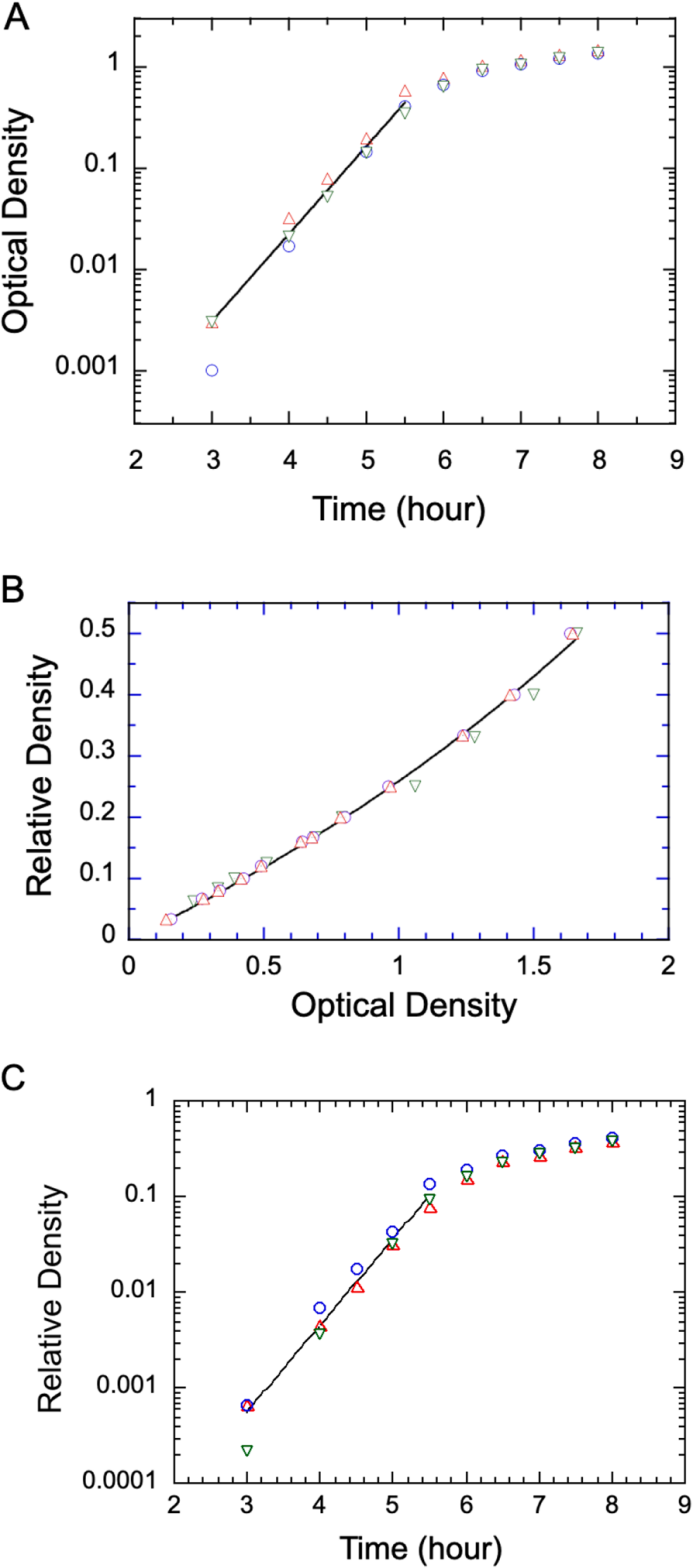
Determination of bacterial growth rate from optical density (OD) measurements and their conversion to relative density (RD) following the application of a measured calibration curve. **A** Three sets of measured OD data shown in the range between 3 and 8 h following initiation of a regrowth (from the right curves in Fig. 1). The line segment represents the fit to exponential growth between 3 h and 5.5 h, yielding bacterial doubling time of 21 ± 3 min. **B** Three sets of dilutions to obtain relative density (RD) values versus the corresponding measured optical density (OD) values. A conversion function of Eq. (2) is fit to the data pulled from all measurements, yielding $\:a=1.34\pm\:0.13$ and $\:b=6.17\pm\:0.47$. The fit curve is shown as the solid line running through the data. **C** The OD data were converted to RD values using the fit formula obtained in B, and the latter were plotted also as a function of time. The line segment shows a similar fit to an exponential growth between 3 and 5.5 h, yielding a doubling time of 20 ± 3 mins

### The relative density method confirms the reliability of growth rate determination by direct fitting of the OD-time curves in the exponential range

We selected portions of data from the regrowth curves in Fig. 1 to determine the growth rate of SM3 in LB. We made this choice because the measured OD data during the exponential phase of the regrowth were free from the complications of the fresh growth curves, for which a lag phase within the first hour or so affects the data measured in the exponential phase. Out of the five sets of regrowth OD curves, we selected three sets that are in closest agreement and replotted them in the range between 3 and 8 h. Data values of the first two hours were less reliable due to small numbers that are easily affected by instrument sensitivity and sample-to-sample variation. Fitting an exponential function to the three sets of chosen data in the time range between 3 h and 5.5 h (Fig. 2A) yields a rate constant of 1.99 ± 0.31 h, which by applying Eq. (1), converts to a bacterial doubling time of 21 ± 3 min .

We then sought to improve the growth rate determination by applying the procedure of a relative density method, as introduced by Lin et al. [15]. The method was designed to correct for an optical effect that causes OD readings to no longer increase proportionally to the cell concentration at high OD values. One starts the relative density method by preparing a series of dilutions of an overnight bacterial growth, recording their concentrations in fractional values as relative density (RD), and measuring the OD of these samples to produce a conversion curve in an OD-RD plot (Fig. 2B). For good reproducibility, we used the fully saturated overnight growth of the SM3 bacteria as the standard of RD = 1, for which a 1:1 dilution with the LB medium, i.e., a relative density of RD = 0.5, gives a high OD reading of 1.55. We applied the empirical fit function introduced by Lin et al. to OD measurements of three sets of calibration samples. This fit function is given by

2 $$\:RD=\frac{a\times\:OD}{(b-OD)},$$

where $\:a$ and $\:b$ are dimensionless fit parameters. We obtained from the fit $\:a=1.34\pm\:0.13$ and $\:b=6.17\pm\:0.47.\:$The OD values shown in Fig. 2A were converted to RD values and plotted against time in Fig. 2B. The RD-time curves (Fig. 2C) are similar in shape to the OD-time curves (Fig. 2A), and an exponential fit in the same window of time yields a rate constant of 2.08±0.36 h^−1^, which converts to a bacterial doubling time of 20±3 min using Eq. (1). Thus, the correction resulted in no significant change in cell doubling time. Although initially surprised, we found the result reasonable, judging by the shape of the RD-OD conversion curve. The reason is that the deviation from the proportional relationship between OD and the actual bacterial concentration is insignificant in the exponential growth phase (OD<0.5), which is the only range of data based on which doubling time is determined. Nevertheless, the same result after applying this calibration and conversion procedure compared with directly fitting the OD-time curves confirms the reliability of the bacterial doubling time we obtained. This consistency also offers assurance to most researchers who do not perform the relative density procedure.

### Doubling time of *E. coli* RP437 from optical density measurements and the relative density method

**Fig. 3:**
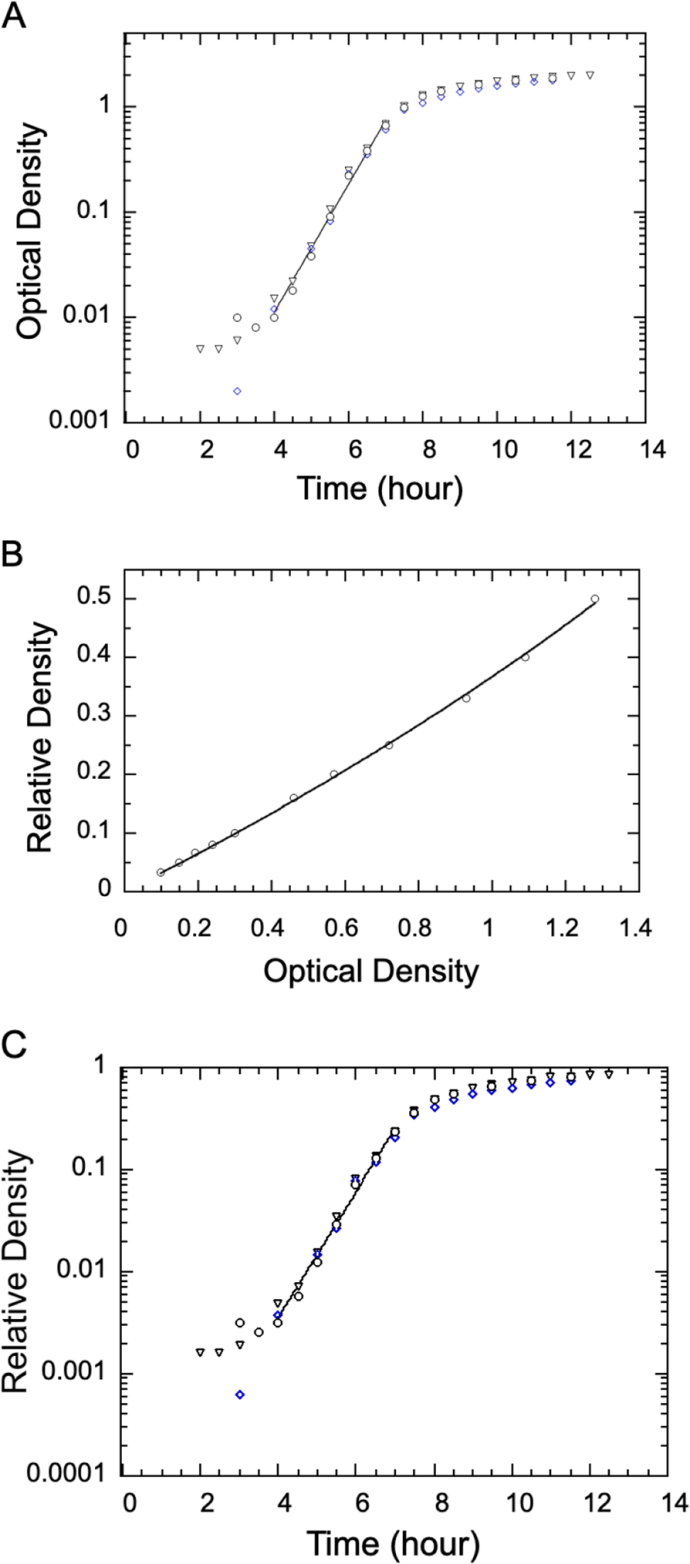
Determination of *E. coli* growth rate from optical density (OD) measurements and their conversion to relative density (RD) following the application of the measured calibration curve. **A** Three sets of measured OD data following the growth procedure described in the main text. The line segment indicates the fit to exponential growth from 4 to 7 h, yielding an exponential rate of 1.40 h^−1^, which converts to a doubling time of 30 min. **B** Three sets of dilutions to obtain relative density (RD) versus their measured optical density (OD). A conversion function of Eq. (2) is fit to the data pulled from all measurements, yielding $\:a=2.40\:$ and $\:b=7.10$. The fit curve is shown as the solid line running through the data. **C** The OD data were converted to RD using the fit formula obtained in B, plotted also as a function of time. The line segment shows a similar fit to exponential growth from 4 to 7 h, yielding a doubling time of 29 mins

We followed the same procedure to measure the doubling time of the RP437 strain of *E. coli*, which grows significantly slower than *SM3*. The general shape of the growth curves, as shown in Fig. 3, is similar to those for the growth of SM3 (Figs. 1 and 2).

From the OD data of both SM3 and *E. coli*, the conversion to RD-time results in only a modest improvement for both cases. Based on the previous report [15], the relative density method appears more applicable to cultures that grow relatively slowly, with the exponential phase lasting several hours or longer. For the rapidly growing SM3, we conclude there is no need for OD-RD conversion in the subsequent measurements in this report.

We also varied the growth conditions to ensure that the growth rate of SM3 does not depend heavily on the concentration of nutrients in the growth medium. Specifically, we varied the nutrient level in LB from $\frac13$ to 3 times the concentrations of bacto-tryptone and yeast extract (with the NaCl concentration kept unchanged at 5 g/L) and found that the growth rate varied by less than 20% (data not shown).

### Measuring shifts in the OD versus time curve of set dilution factors offers an independent approach to determining the bacterial doubling time

**Fig. 4:**
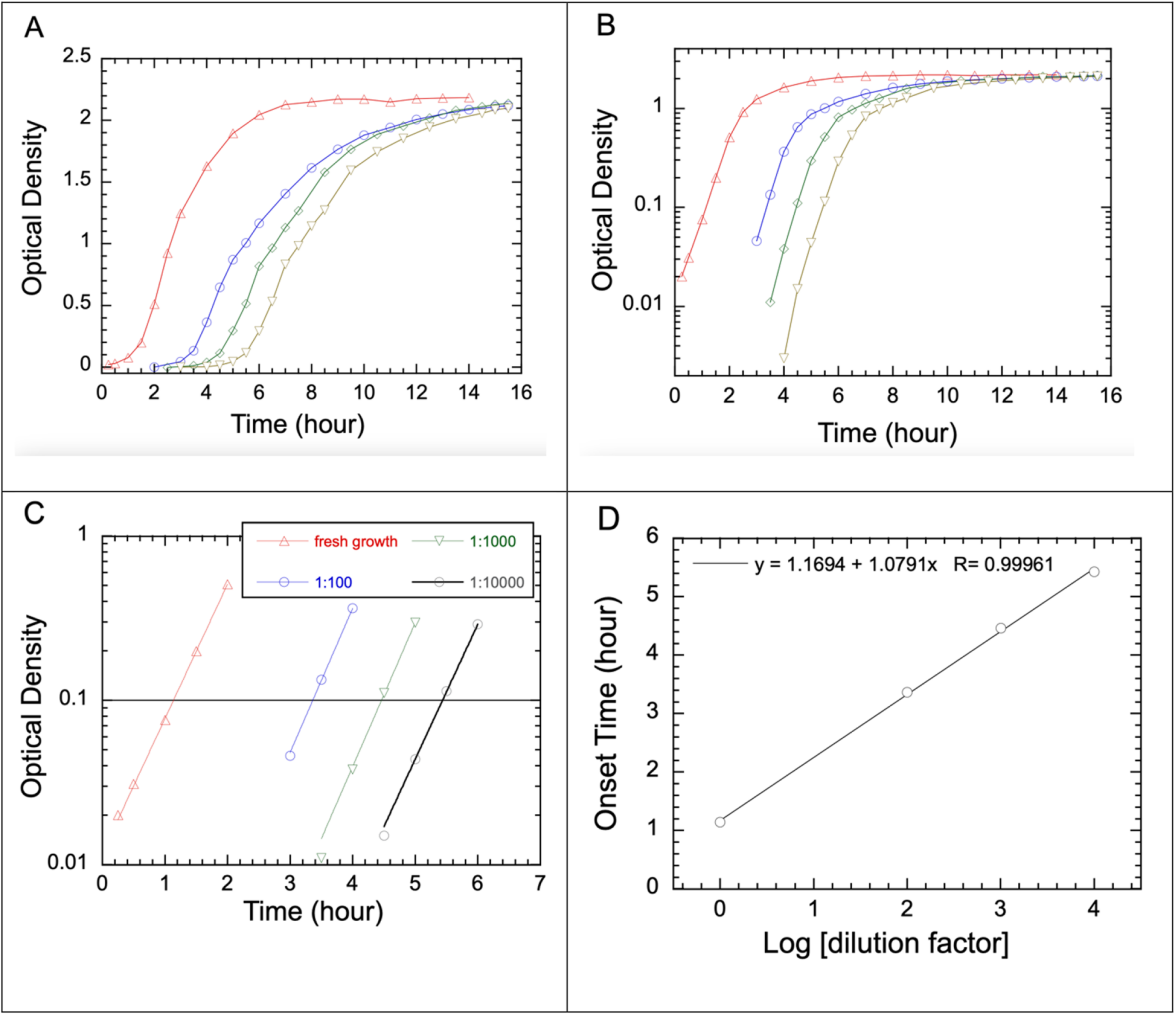
Determination of doubling time for SM3 from the shifts in the growth curve of set factors of dilutions. **A** Fresh growth of *SM3* and subsequent regrowth following one-hour activation with dilution factors of 100x, 1000x, and 10000x. **B** The same data as in A, but shown in log-linear plot instead of linear-linear plot. **C** Selected data from A or B in the range of exponential growth, with exponential fits for fresh growth, followed by dilutions of 100x, 1000x, and 10000x. Intercepts of all four fit curves with OD = 0.1 are obtained and noted as the onset time. **D** The onset time plotted as a function of the logarithm of the dilution factor. The bacterial doubling time is obtained from the slope of the fit to be 1.079 h. The bacterial doubling time was obtained to be $\:{t}_{D}=0.325$ h or 19.5 min, using Eq. (6) under Methods

The nature of exponential growth in a fresh liquid culture allows us to design another simple method of measuring bacterial doubling time. Aside from using the OD curve of a fresh growth, we diluted the fresh growth sample of SM3 by 100x, 1000x, and 10000x to measure the growth curves of samples at these known factors of dilution. From the temporal shift ($\:{t}_{shift}$) between the consecutive OD curves that differ in dilution by a factor of ten (Fig. 4), the doubling time can be calculated as shown in the Methods section, under “Shift analysis of serial dilution for doubling time determination.” Applying a fit of the onset time versus the exponent of the dilution factor, in the power base 10 (Fig. 4D), a doubling time of 19.5 min is obtained. This value is practically the same as those obtained from fitting the OD or RD versus time data. This method is convenient and reliable for determining the bacterial doubling time, as long as samples can be made with accurate factors of dilution. The validity of the bacterial doubling time thus determined only depends on temporal shifts of their growth curves and is unaffected by the shortness of the exponential growth phase.

### The colony-forming unit method offers reliable determination of doubling time of live bacteria

To confirm the growth rate of bacteria determined by optical density measurements, we decided to count the number of live bacteria using the conventional colony-forming unit (CFU) method. When dormant SM3 in overnight growth are mixed with fresh LB medium and shaken at 37^o^C, they activate immediately, resume growth, and start dividing in about one hour. The growth in the density of viable bacteria was measured by diluting aliquots of a fresh growth at several points of time and counting the CFUs, from which the bacterial doubling time can be determined.

**Fig. 5:**
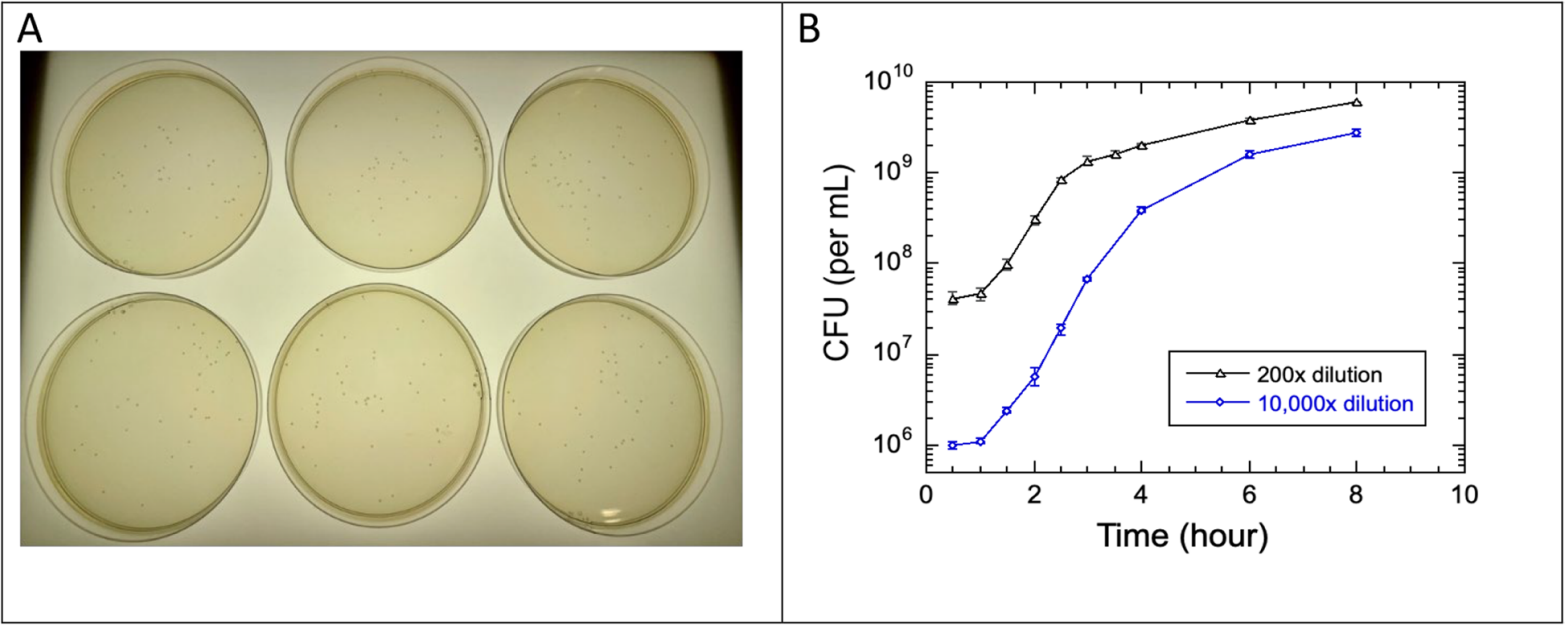
Measurement of growth of SM3 by the method of counting colony-forming units (CFUs). **A** SM3 colonies visible on six 1% agar-LB plates. The CFUs are counted for all colonies on each plate. These six plates were prepared under the same conditions that were placed on a projector screen and imaged by a cell phone camera (iPhone 12, Apple Inc.). **B** CFU counts over time for growth of SM3 initiated by diluting an overnight culture into fresh LB medium with two different factors. Graphs are shown on a log-linear scale for the two dilution factors: 1:200 and 1:10,000. Both curves are similar in shape, but they differ in initial starting concentration of cells. Both curves show a lag phase, an exponential growth phase, and the beginning of a stationary phase. The error bars, some smaller than the size of the symbols, indicate standard errors. Assuming that the final cell concentration at the stationary phase reaches the same as that of the saturated overnight growth, it is estimated to approach 1.0x10^10^ cells per milliliter. This estimate was obtained using the first value on the left of either curve, multiplied by their respective dilution factors

 The growth rate and thereby the cell doubling time were directly calculated based on the CFU counts during the exponential phase of the growth (Fig. 5). The exponential growth phase lasted only about two hours, followed by a gradual slowdown in the growth rate or the rate of cell division. The growth rate slowdown depended on the cell density, suggesting it may result from depletion of nutrients, such as utilizable carbon [6], or from the secretion of self-inhibiting factors through a mechanism of quorum sensing [26]. The primary mechanism of a slowdown in cell division rate may be identified by additional experiments over a wider range of dilution factors, but the purpose of them goes beyond the scope of this study.

The curve measured for the 1:10,000 dilution (Fig. 5B) covers a full range of the bacterial growth that includes a lag phase, a 3-hour-long exponential growth, and a gradual approach to saturation. The data for this set of measurements are listed on Table 1 for convenient fitting with various growth models (see Additional Discussions).

**Table 1:** Growth in bacterial density, measured by counting colony forming units (CFUs)

Time (hour)	Dilution factor	Plates counted	Average colonies	Std err (colonies)	Cell density (bacteria/ml)	Std err (bacteria/ml)
0.5	10^5^	9	10.1	0.89	1.01 × 10^6^	0.89 × 10^5^
1.0	10^5^	8^a^	11.0	0.68	1.10 × 10^6^	0.68 × 10^5^
1.5	10^5^	9	24.1	1.70	2.41 × 10^6^	1.70 × 10^5^
2.0	10^6^	9	5.78	1.26	5.78 × 10^6^	1.26 × 10^6^
2.5	10^6^	9	19.2	2.52	1.92 × 10^7^	2.52 × 10^6^
3.0	10^6^	9	67.4	3.76	6.74 × 10^7^	3.76 × 10^6^
4.0	10^7^	9	39.1	2.71	3.91 × 10^8^	2.71 × 10^7^
6.0	10^8^	8^a^	16.0	1.26	1.60 × 10^9^	1.26 × 10^8^
8.0	10^8^	6^b^	27.5	2.55	2.75 × 10^9^	2.55 × 10^8^

We did not perform an additional set of experiment for this time point alone, as the standard error was already below 10%. The experiments were performed with three repeats, in triplicates

^a^1 plate failed to yield reliable counts

^b^One set of 3 plates for hour 8 failed

### The OD and CFU measurements yield consistent values of cell doubling time

**Fig. 6:**
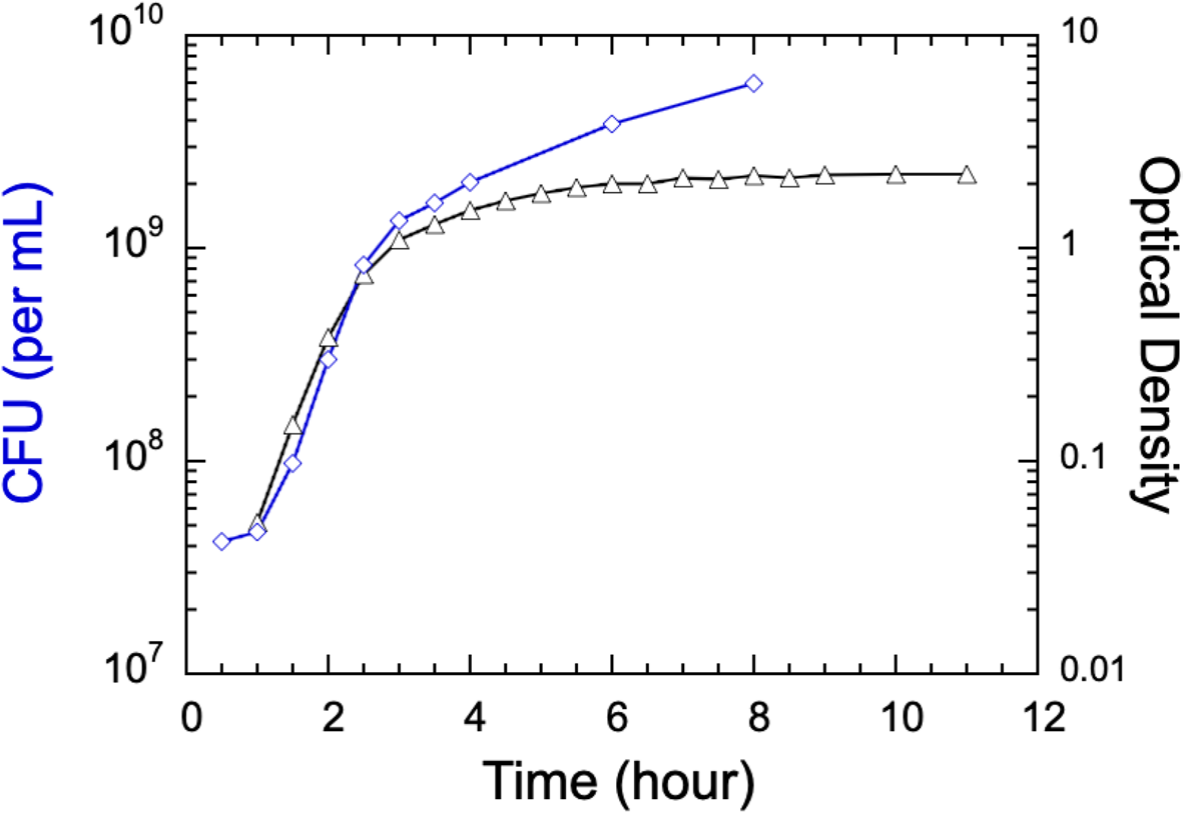
Comparison between plots obtained by both the CFU cell count (left vertical axis) and the OD measurement (right vertical axis). These two methods show similar exponential growth over 2 to 3 h, yielding consistent doubling times, i.e., 21 min from the CFU curve compared with 21 ± 3 min from the OD measurements (Fig. 2). The CFU data also show that cell numbers do not increase in the first hour. This lag phase of about one hour could not be determined by the optical density measurement due to the instrument sensitivity limit at low OD values

A close comparison between the growth data measured as OD and CFUs confirms the consistency of cell doubling times determined by these two independent methods. The plot in Fig. 6 shows a typical OD curve over eleven hours (black curve) compared to the average CFU numbers over an 8-hour period (blue curve). The values of the two sets of data are labeled on the left for CFUs and on the right for OD. A simple exponential fit to four data points of 1.0, 1.5, 2.0 and 2.5 h of the CFU data yields a growth rate of k = 1.96 h^−1^, which converts to a doubling time of 21 min. This doubling time is in full agreement with the doubling time obtained above from the OD measurements. Interestingly, the CFU plot shows that SM3 cell division persists over several hours when the OD curve has plateaued, confirming that the OD values in the range of OD > 0.5 are no longer proportional to the number density of SM3.

## Additional discussion

An open issue in microbiology is the large range of variation among the bacterial growth rate reported in the literature, due to the variety of techniques used. Reasons for reliability issues in literature reports of cell growth rates include species- and strain-dependance; variability of medium and growth conditions (temperature, aeration, etc.); the narrow range of the exponential phase; an arbitrary choice of OD range for exponential fitting; and, for some species, a change in growth behavior following repeated cycles of growth. Thus, there are still no commonly accepted values for growth rates for even the most extensively studied bacteria, such as *E. coli*, *S. aureus*, and *P. aeruginosa* (see Table 2, and reference therein). For these and many other fast-growing bacteria, the variation in reported values of their growth rate is often as large among the various mutant strains of the same species as among different species, making it hard to reliably compare them. Therefore, the responsibility lies with researchers to specify the exact strains grown under what exact conditions. In addition, efforts to report reliable growth rates must be expanded far beyond extracting the values from a few growth curves to report an average rate with a seemingly sensible error bar.

**Table 2:** Growth rate and doubling time of selected species of bacteria

Species	Strain	Growth medium^a^	Method of determination	Growth rate (1/hr)	Doubling time (min)	Reference
SM3		LB	optical density (OD)	1.99±0.31	21±3	this work
		LB	relative density (RD)	2.08±0.36	20±3	this work
		LB	serial dilution & OD	ND	19.5	this work
		LB	colony forming unit (CFU)	1.96	21	this work
E. coli	RP437	LB	OD	1.40	30	this work
	RP437	LB	RD		29	this work
	ATCC 25922	MHB	CFU	0.49±0.03^b^		[27]
	MG1655	LB	OD		20.9±0.3	[28]
	B/r	LBG	OD and Coulter Counter		23	[29]
	DH5α	LB	OD and CFU	1.25±0.02		[30]
S. aureus	LAC91	TSB	OD		22.4±2.1	[31]
	KH38	TSB	OD		33.4±3.0	[31]
	HH49	TSB	OD		38.0±5.4	[31]
	HH36	TSB	OD		24.9±1.8	[31]
	ATCC29213	MHB	CFU	0.45±0.03^b^		[27]
	NCTC8325-4	MHB	CFU		57±4.9	[32]
	TCH1516	CA-MBH	OD	1.12±0.08		[33]
	TCH1516	RPMI	OD	0.75±0.1		[33]
	8325-4	ISB	CFU		37.5±1.0	[34]
P. aeruginosa	PAO1	TSB	OD	0.265	157.2±19.4	[31]
	PAO1 & PA14	LB	OD		24-27	[35]
	CF isolates	LB	OD		50-74	[35]
	ATCC 27853	MHB	CFU	0.67±0.03^b^		[27]
	PAO1	FAB, NY	OD		59±0.1	[36]
	PAO1	FAB+KNO_3_	OD		92±4.7	[36]
	PAO1	LB				[37]
	PAO1	LB	OD	0.29±0.02		[38]

^a^*LB* Luria-Bertani broth, *MHB* Muller Hinton Broth, *LBG* Luria-Bertani broth + 0.4% glucose, *TSB* Tryptic soy broth, *CA-MHB* Cation-adjusted Muller Hinton broth, *RPMI* Roswell Park Memorial Institute, *ISB* Iso-Sensitest Broth, *FAB* Ferric Addition Buffer, *NY* Nutrient broth-yeast extract

^b^Obtained from fit to modified logistic function

An additional complication in determining the growth rate of bacteria is the variety of models that have been applied to fit bacterial growth curves [8, 30, 39–42]. Many models have been developed, particularly in connection with food spoilage or preservation [1, 8, 9, 40, 42, 43]. Most models, including those that are rather analytically complicated, are not rooted in robust principles. The modified logistic function [27, 42], for instance, is empirical in nature. By capturing both an exponential growth phase and an asymptotic approach to saturation towards a high concentration limit, the modified logistic function is used extensively for fitting bacterial growth curves to yield their growth rate. More advanced analytical models, such as the modified Gompertz model [40] and the Baranyi-Robert model [8, 39, 43], have all required simplifying assumptions in their derivations. We tried to fit these three models to our selected dataset, listed in Table 1 and shown in Fig. 5B for the 10,000x dilution curve, only to find out that whereas the logarithm of our measured cell density data versus time fits well the modified logistic function [41], neither the Gompertz Eq. [1], nor the Baranyi-Robert equation [8] fits our data over the entire range. Note some rate constants listed in Table 2 were obtained from fits to the modified logistic function, whereas  the majority of values in the table were determined through the simplest exponential fit to a narrow range of growth in time, casting some doubt on their reliability. A comprehensive comparison of fit models goes beyond the scope of this report.

The close comparison of the growth rates between SM3 and *E. coli* RP437 under the same conditions determined that SM3 indeed proliferates faster than this well-studied wildtype strain of *E. coli*, which is known to be fast-growing. This result is interesting from the viewpoint of gut physiology. The bacterial metabolic rate is limited by various regulatory processes in transcription, protein expression, and the mechanics of physical division. The growth rate of SM3 appears to be among the highest that we know of. This makes sense, given its origin from the temperate and nutrient-rich animal intestines.

One limitation of this study is its narrow focus on determining the growth rate of SM3 in a standard culture. The comparison with other species has been limited to only one strain of wildtype *E. coli*. We set an aim to avoid reporting the growth rate of a new bacterial species measured by only a few sets of measurements using a single method or two. Whereas reliable comparison of growth rates across bacterial species may be the shared goal among microbiologists, this study offers some insights on why the reported growth rates of most species of bacteria are highly variable.

When using optical density measurements, one must be aware that the relationship between OD and cell density may be complicated by changes in the cell size and shape during growth. This complication is most acute within the first hour or two of a fresh growth, often referred to as the lag phase or activation phase. After the stationary state bacteria are infused into a fresh medium, they become activated and resume growth within minutes [5]. This growth manifests as increased cell diameter and length. Depending on the species, the first cycle of division may take over an hour to occur, whereas subsequent rounds of division occur in much less time [17]. It is the cell doubling time in later rounds of division that most studies seek to determine. This doubling time is inversely proportional to the bacterial growth rate. Therefore, the proper window of time for determining the growth rate is after the lag or activation phase, at which point the number of cells begins to grow exponentially, but before the rate of growth decreases and later plateaus.

The plateau on the bacterial growth curve may be due to the bacteria’s consumption of a significant fraction of the available nutrients [6]. It may also be due to accumulation of metabolic products or waste [16], which bacteria detect in the form of quorum sensing [26]. If better instrument sensitivity is feasible, the fresh growth ought to be initiated at as low a density as possible, allowing a long enough duration of growth within the exponential phase. Due to the instrument detection limit at the lower end, however, the exponential portion of individual growth curves is typically rather short. Since measuring time shifts in growth curves only require intercepts of these growth curves at a chosen OD value, say 0.1 (Fig. 4), the curve shifting method introduced in this study is advantageous compared to relying on fitting exponential growth curves directly. This curve shifting method is similar to the SGT method published previously [14]. Both methods rely on the exponential nature of the bacterial growth. One advantage of the curve shifting method is that the exponential portion of the curve can be as short as an hour while sufficing to reliably determine the doubling time.

The colony forming unit assay has withstood the test of time as the most reliable method to measure bacterial growth rate. Still, care must always be taken to minimize the errors introduced by dilutions. Multiple agar plates have to be used in order to average out stochastic errors when counting variable numbers from plate to plate. The dilution factors ought to be designed such that colony counts on agar plates are large enough to minimize standard deviations, while ensuring a low probability that two live bacteria happened to settle so close to each other on the plate that they only formed one discernable colony spot. The latter case would result in an underestimation of cell density [19]. The practical range is from tens to a few hundred cells per 8-cm diameter plate, but the incubation time must also be limited so that colonies do not grow too large, increasing the frequency for some to overlap and fuse.

## Conclusion

This report offers an example of validation of determinations of bacterial growth rate using several methods based on optical density and colony count measurements. Our data show that the doubling time in LB at 37^o^C for *Enterobacter* sp. SM3 is 21 min, compared with 29 min for *E. coli* RP437. Thus, the recently identified enterobacterium species SM3 grows faster than *E. coli*, based on the head-on comparison of this study. The results of this study suggest that SM3 is among the fastest in growth among species of cultured bacteria. Our study also conveys the need for consistency verification among multiple techniques to avoid reporting variable values of doubling time for particular species or strains of bacteria, as seen in the literature.

## Methods

### Preparation of bacterial culture

The *Enterobacter sp. SM3* was isolated from the feces of mice suffering from inflammation [24]. The strain was gifted from Dr. Sridhar Mani of Albert Einstein College of Medicine. *E. coli* RP437 strain (HCB 33) was gifted from the Howard Berg laboratory, Harvard University. Both species of bacteria were stored at -80^o^C in grown culture mixed with 30% glycerol. To start a fresh growth, a sterile inoculation loop was used to scratch a frozen stock and inoculate a flask of LB (Lysogeny Broth: 10 g/L tryptone, 5 g/L yeast, and 5 g/L NaCl, dissolved in deionized water and autoclaved). To vary the nutrient level in LB, media were made with $\frac13$ or 3 times the concentrations of bacto-tryptone and yeast extract but with the NaCl concentration kept unchanged, at 5 g/L.

Bacteria were grown at 37^o^C in an incubator shaken at 160 rpm. Prior to starting subsequent growth by dilution, a flask with fresh LB was prewarmed in the incubator for 30 mins so that the cell growth consistently took place at 37^o^C.

### Determining growth rate by measuring optical absorption

We applied three methods of determining the bacterial growth rate using optical absorption. First, we measured the attenuation of light transmitted through a 1-cm sized cuvette (Fisher Scientific) holding a bacterial suspension, which we took from the incubator at different time points of growth. The measured quantity is called optical absorption or optical density (OD), which is the logarithm of the ratio of incident light to transmitted light. It was read directly off a spectrophotometer at 600 nm (Shimadzu). We plotted the OD values versus time in the form of a log-linear plot so that we could visually identify the period of exponential growth (following a latent or lag period of 1 h or so). We determined the doubling time from the rate of the exponential fit by using t_d_=ln(2)/k, where k is the growth rate [1].

The second and third methods both stem from the OD measurements. Because of the intricacies of these methods, we describe them in the following as separate sub-sections.

### Shift analysis of serial dilution for doubling time determination

A serial dilution method was carried out by measuring the OD curves following two sets of dilutions. First, we diluted 100 µL of overnight growth in 20 mL of fresh LB  and called the mixture fresh growth. We measured the OD of the fresh growth over the next several hours. One hour later, we added 200 µL, 20 µL, and 2 µL aliquots of fresh growth into flasks of 20 mL LB, resulting in dilution factors of 100, 1000, and 10,000, respectively. We then measured the OD of those samples over their growth for up to 15 h. We analyzed the shifts of OD curves to determine the doubling times. Recognizing that the growth curves change most rapidly during the exponential phase, we fitted the data in the exponential phase and obtained the intercept of each fit curve with OD = 0.1. We noted the value as the onset time, $\:{t}_{onset}$ (Fig. 4C).

Here is a simple derivation to prove that the onset time increases linearly with the logarithm of the factor of dilution. We first define mathematically the shift time, T_s_, as the time interval between the onset time of a certain factor of dilution and that of the initial growth curve. Thus,

3 $$\:{T}_{s}={t}_{onset}-{t}_{o}$$

where $\:{t}_{onset}$ is the onset time of a growth curve of any factor of dilution, and $\:{t}_{o}$ is the onset time for the initial growth curve. The nature of exponential growth dictates that the shift time $\:{T}_{s}$ corresponding to a dilution factor of 10^N^ relates to the doubling time $\:{T}_{d}$ according to the following relationship:

4 $$\:\:{10}^{N}={2}^{\frac{{T}_{s}}{{T}_{d}}}.$$

Taking the logarithm on both sides and rearranging yields

5 $$\:{T}_{s}=\left(\frac{{T}_{d}}{log\:2\:}\right)N.$$

Combining Eq. (3) and Eq. (5) yields

6 $$\:{t}_{onset}={t}_{o}+\left(\frac{{T}_{d}}{log\:2\:}\right)N\:.$$

Here, $\:\frac{{T}_{d}}{log\:2\:}$ is the slope in the $\:{t}_{onset}$ versus N plot, as shown in Fig. 4D. Thus, this method yields $\:{T}_{d}$ from the simple expression for the slope, which is found by a linear fit, as shown in Fig. 4D.

### Determining doubling time from relative density data

We followed a relative density method as described in detail by Lin et al. [15]. Briefly, we started with an overnight (16 h) growth of *SM3* as the standard density of unity. By diluting it, we yielded a series of samples of fractional relative density (RD). By measuring the OD of samples of fractional relative density values, we obtained a conversion curve in the form of an RD-OD plot. We then measured the OD values of samples of unknown cell density and converted them to RD values that were reliably proportional to cell density. Accordingly, fitting the exponential part of the RD versus time plot is more reliable in determining the cell growth rate than directly fitting the OD versus time plot. Note in this method the absolute density of cells is not needed to determine the growth rate or doubling time; the relative density values suffice [15].

### Measuring density of live cells by counting CFUs

We followed the established method of counting colony-forming units (CFUs) to determine the density of live bacteria [19, 20, 22]. At each time point of the bacterial growth in a flask of LB, that is, every 20–30 min, a small aliquot was taken. We serially diluted this aliquot in fresh LB and spread 100 µL of the resulting sample over a 1% agar plate by tilting and turning the plate. Plates were incubated at 37 °C overnight  and then observed when the colonies grew to visible spots. Examples of plates with visible colonies are shown in Fig. 5A. The factors of dilution, ranging from 10^3^ to 10^6^, were chosen based on the time point and the estimated density of cells in the growth culture so that numbers of colonies on the order of 10 s or 100 s could be counted on an entire plate. This technique is tedious but reliable, as long as proper dilution factors were made so that each plate has enough colonies but not too many.

## Data Availability

All data generated or analyzed during this study are included in this published article.

## Abbreviations

LB: Lysogeny broth
OD: Optical density
CFU: Colony forming unit
DSS: Dextran sodium sulfate
IBD: Inflammatory bowel disease
SGT: Start of growth time
RD: Relative density
